# ‘Pediatric Bipolar Disorder’ rates are still lower than claimed: a re-examination of eight epidemiological surveys used by an updated meta-analysis

**DOI:** 10.1186/s40345-021-00225-5

**Published:** 2021-06-25

**Authors:** Peter Parry, Stephen Allison, Tarun Bastiampillai

**Affiliations:** 1 College of Medicine and Public Health, Flinders University, Adelaide, Australia; 2 Children’s Health Queensland Clinical Unit, School of Clinical Medicine, University of Queensland, Brisbane, Australia; 3 Mind and Brain Theme, South Australian Health and Medical Research Institute, Adelaide, Australia

## Abstract

**Background:**

‘Pediatric bipolar disorder’ (PBD) is a controversial diagnosis with varying rates of clinical diagnosis. A highly cited meta-analysis (Van Meter et al. 2011) of a dozen epidemiological surveys suggested a global community prevalence of PBD of 1.8%. This was updated to 3.9% with eight additional surveys (Van Meter et al. 2019a). In terms of the Cochrane Handbook for Systematic Reviews of Interventions, the heterogenous community surveys were arguably unsuitable for statistical meta-analysis and warranted a narrative analysis. A narrative analysis (Parry et al. 2018) of the original 12 surveys concluded rates of PBD were substantially lower than 1.8% and led to a nine-article debate on the validity, arguable overdiagnosis and iatrogenic aspects of the PBD diagnosis (e.g. Carlson and Dubicka Child Adolesc Mental Health 21:86–87, 2019). This article extends the narrative analysis to include the eight newer community surveys.

**Methods:**

A narrative analysis of the methodologies and the prevalence rates reported by the epidemiological surveys.

**Results:**

Across all twenty surveys there was significant variation in methodologies and reported prevalence rates. Of the eight newer surveys, five (two Brazilian, one English, one Turkish, one United States) provided information of pre-adolescent prevalence rates of bipolar spectrum disorder. These pre-adolescent rates were zero or close to zero. Rates of adolescent hypomania and mania were higher, but follow-up data in two studies suggested hypomania might sometimes achieve prolonged remission or not lead to adult bipolar disorder.

**Limitations:**

Methods in the original surveys vary and criteria used for various bipolar diagnoses were not always fully described. This limitation applies to a narrative analysis but also to a statistical meta-analysis.

**Conclusion:**

Bipolar disorder is very rare in childhood and rare in adolescence. PBD as a diagnostic construct fails to correlate with adult bipolar disorder and the term should be abandoned. Hypomanic syndromes in adolescence may not always progress to adult bipolar disorder. Early diagnosis of bipolar disorder is important, but over-diagnosis risks adverse iatrogenic consequences.

## Introduction

### The ‘pediatric bipolar disorder’ (PBD) hypothesis

The prevalence rate of bipolar disorder (BD) in children and adolescents has been hotly debated since the 1990s (Biederman 1998; Klein et al. 1998). The most controversial aspect has been whether BD affects pre-pubertal children in substantial numbers or whether the first hypomanic/manic episode is almost invariably post-puberty. The debate matters because either young children need pharmacotherapy for BD or such pharmacotherapy poses unwarranted iatrogenic risk.

The former view presupposes that mania presents differently in children with one hypothesized PBD phenotype involving ultradian cycling of brief mood episodes shorter than one day (Geller et al. 1995; Axelson et al. 2011) or alternatively, as chronic irritability generally without mood elevation (Wozniak et al. 1995). The chronic irritability PBD phenotype was later subsumed under the new DSM-5 diagnosis of disruptive mood dysregulation disorder (DMDD) (Krieger et al. 2013).

In contrast, the ‘classical’ or historical view since the time of Kraepelin (1921), posits that hypomanic/manic episodes typically first manifest from mid-adolescence into young adulthood. True mania in pre-adolescent children occurs but is very rare (NICE 2014).

The PBD hypothesis found traction in several US academic institutions and was translated into US clinical practice to become the most common diagnosis on pre-adolescent inpatient units (Blader and Carlson 2007). In contrast, apart from a small number of academic units in other countries (Parry et al. 2019b), the classical view held sway internationally.

### Discrepancies in international rates of clinical BD diagnoses in children and youth

This academic divergence of views has been reflected in inpatient discharge diagnosis rates where the US has had several-100-fold higher rates of BD diagnosis amongst children and young adolescents than in other nations. For example: In the 5–9 age group, the US rate was 27 per 100,000, whereas it was 0.22 in New Zealand, 0.14 in Australia, 0.03 in Germany, and 0.00 in England; in the 10–14 age group, the US rate was 134 per 100,000, while 3.9 in Australia, 1.3 in New Zealand, 0.48 in England, and 0.46 in Germany (Clacey et al. 2015). A study of 5,483 under-18-years-old inpatients discharged between 1997 and 2014 by two Prague psychiatric units in the Czech Republic identified 46 patients with DSM-IV BD (93% BD-I, 7% BD-II), the youngest first manic episode was 11.5-years-old (Goetz et al. 2015). A survey of 64 of Finland's 69 pediatric psychiatric inpatient units in January 2000 found 504 children and adolescents ages 5—18-years-old of whom only eight had a BD diagnosis, a rate of 1.6% of a high acuity cohort (Sourander, 2004). A comprehensive review (Kessing et al. 2014) of all outpatient as well as inpatient under-19-years-old BD diagnoses in Denmark between 1995 and 2012 found 346 cases, the annual clinical prevalence rate amongst all Danish youth was 0.001% in the first eight years and increased to 0.002% to 0.004% during the latter eight years of the survey period and "only 25% of the patients were below 16.2 years of age when the diagnosis of mania/bipolar disorder was made for the first time" (p. 6). A comparison of BD diagnoses for ages 0–19-years between 500 US psychiatric inpatient units and all NHS psychiatric inpatient units in England from 2000 to 2010 found no pre-adolescent diagnoses in England and there were more BD diagnoses in the US by age 5 than by age 19 in England (James et al. 2014).

This wide discrepancy in child and early adolescent BD diagnoses is also reflected in six longitudinal high-risk offspring of parents with BD studies (Canadian, Dutch, Swiss, US Amish, US multisite University of Indiana, US Pittsburgh), where only the Pittsburgh study that employs the PBD hypothesis found “in those offspring who developed BD, 50% had mania prior to age 12 (compared to 0% in other studies)” (Duffy et al. 2017: p. 6). The Pittsburgh study (Axelson et al. 2015) reported: “Of the 15 high-risk offspring who had a manic episode, five (33%) had the first episode before age 10 and eight (53%) before age 12, with the earliest at 8.1 years of age”, although they found a slightly older mean age of onset for hypomania (13.4, SD = 3.8) and bipolar spectrum disorder (12.1, SD = 4.0) (p. 642).

### Prior re-examination of first meta-analysis of ‘PBD’ community prevalence

A highly cited meta-analysis of 12 epidemiological studies (Van Meter et al. 2011) claimed a global community prevalence rate of PBD of **1.8%.** However, our narrative analysis of each of the 12 studies (Parry et al. 2018) noted: heterogeneity of methodologies arguably made them unsuitable for meta-analysis; subjects were mostly adolescents, and informant responses poorly correlated. We concluded (Parry et al. 2018):

[T]he 12 studies suggest that where methodology correlated parent and child reports for agreement, and included impairment criteria, that rates of bipolar-I disorder in children and adolescents were close to zero outside the United States and only slightly higher in the United States, though rates of bipolar spectrum disorder were slightly higher. Articles that cite the meta-analysis need to critically examine the original studies. (p. 21)

In an accompanying editorial, Carlson (2018) commented on our reanalysis:

[R]ates of mania/bipolar I disorder are lower in both places than referenced by Van Meter, et al. Lifetime rates in most adolescents within the United States are between 0% and 1% and outside the United States between 0% and 0.1%. Bipolar spectrum definitions were inconsistent. (p. 23)

Van Meter and colleagues responded to our reanalysis, which prompted a nine-article debate section in *CAMH* (Carlson and Dubicka 2019; Duffy 2019; Goldstein et al. 2019; Hazell 2019; Hillegers 2019; Pan et al. 2019; Parry et al. 2019a; Stringaris 2019; Van Meter et al. 2019b). Duffy, lead researcher for the longitudinal Canadian high-risk offspring study, summarised research data and concluded (Duffy 2019):

The elephant in the room is that there is no evidence from high-risk or clinical longitudinal studies or from imaging or genetic studies to support that this paediatric bipolar phenotype has anything to do with adult bipolar disorder. (p. 100)

Stringaris (2019) lamented that in the UK the idea of a child having BD was usually dismissed. However, citing Lohr et al. (2015) regarding high rates in Kentucky of antipsychotic prescribing (based predominantly on BD diagnoses) to “the younger than 7-year-olds” and Ray et al. (2019) regarding the "4.29 hazard ratio for death” due to the metabolic and cardiotoxic effects of these agents when daily dose is above 50 mg chlorpromazine equivalents (e.g. 1.5 mg risperidone, 2.5 mg olanzapine), he criticized the likely “devastating consequences” of PBD overdiagnosis in the US (p. 106). Stringaris noted that 70% of the prescriptions for the 2.4% of 6-year-olds in Kentucky were written by non-child psychiatrists.

### Updated meta-analyses’ community prevalence estimates of PBD

The International Society for Bipolar Disorders (ISBD) Task Force on PBD updated Van Meter et al. (2011)’s meta-analysis by using (Goldstein et al. 2017): “an identical search strategy [to] identify six new studies” (p. 525): two from Brazil (Anselmi et al. 2010; Pan et al. 2014), and single studies from the US (Roberts et al. 2007), Germany (Tijssen et al. 2010), Canada (Kozloff et al. 2010) and Sweden (Päären et al. 2014). By combining all 18 studies the ISBD Task Force (Goldstein et al. 2017) reported a total study population of: “31,443 youth aged 7–21 years, 576 of whom met criteria for bipolar spectrum disorders” and by applying meta-analysis they found an “updated weighted average prevalence of bipolar spectrum disorders [of] **2.06%** (95% CI 1.44%-2/95%)” (p. 526).

Most recently, Van Meter et al. (2019a) further updated the meta-analysis with two additional epidemiological studies: from the UK (Vizard et al. 2018), and from Turkey (Karacetin et al. 2018). They also deleted the New Zealand study (Kim-Cohen et al. 2003) from the original twelve as it was unclear what the under-18 age data was on BD. From this latest therefore 19-study meta-analysis Van Meter et al. (2019a) found the “weighted average prevalence of bipolar spectrum disorders was **3.9%** (95% CI, 2.6%—5.8%)” (p. e1), more than double their 2011 meta-analysis result of 1.8%.

Van Meter et al. (2019a) did not cite the 9-article debate on their earlier meta-analysis, therefore this re-analysis of their updated meta-analysis, particularly in light of the increased predicted prevalence of PBD or BD in the pediatric age range, is to continue this debate as new epidemiological data emerges.

## Methods

### Analyzing data in studies with heterogenous methods and results

We argue that the significant heterogeneity between studies undermines ability to apply meta-analytic statistical analysis. As in our 2018 reanalysis of their 2011 meta-analysis we believe a narrative approach is more scientifically rewarding. As cited in our 2018 reanalysis, the *Cochrane Handbook for Systematic Reviews of Interventions* (Deeks et al. 2011: Part 2, chapter 9.1.4) advises: “If studies are clinically diverse then a meta-analysis may be meaningless, and genuine differences in effects may be obscured. … Further, it is important not to combine outcomes that are too diverse.”

Van Meter and colleagues have undertaken a rigorous internet search for community psychiatric epidemiological studies in the pediatric age range to identify these 19 studies. That is a great service to the field. Much information can be distilled on the question of early-onset and very-early-onset BD, particularly when each study is analysed and compared with others in a narrative framework.

## Results 

Readers can revisit our narrative analysis of the first 12 epidemiological studies (Parry et al. 2018) and these are represented along with the cited prevalence rates from Van Meter et al. (2011) and Van Meter et al. (2019a) in Table 1. In this article we have performed the same qualitative narrative analysis on the newer eight studies.

**Table 1:** The original 12 epidemiological studies reported by Van Meter et al. (2011)

Source study Subjects	Location Year completed Criteria	Instrument Prevalence period Age Informant	Van Meter et al 2011	Van Meter et al. (2019a)			Parry et al. (2018)		
			PBD %	BD-I %	BD-I & BD-II %	Bipolar Spectrum %	BD-I %	Total Bipolar Spectrum %	
Kim-Cohen et al. 2003 N = 973	New Zealand 1985 DSM-III	DISC 12 mth Not asked or Adult age 21	1.8	N/A	N/A	N/A	0 or N/A	0 or N/A	
Verhulst et al. 1997 N = 780	The Netherlands 1993 DSM-III-R	DISC 6 mth 13–18 years Parent & adolescent	2.8 added	1.2 parent	1.2 parent	N/R	1.9 added 0 agreement	2.8 added 0 agreement	
Canals et al. 1997 N = 290	Spain 1994 ICD-10, DSM-IV	SCAN Point 17–18 years Adolescent	2.4 ICD	N/R	0 DSM	N/R	0 DSM 0 ICD	0 DSM 2.4 ICD	
Lynch et al. 2006 N = 723	Republic of Ireland 2002 DSM-IV	K-SADS Lifetime 12–15 years Parent & adolescent	0	0	0	0	0	0	
Benjet et al. 2009 N = 3,005	Mexico City 2005 DSM-IV	CIDI 12 mth 12–17 years Adolescent	2.5	2.0	N/R	2.5	2.05	2.5	
Stringaris et al. 2010 N = 5,326	United Kingdom 2007 DSM-IV	DAWBA Lifetime Parent & adolescent 8–19 years	1.2	N/R	0.1	1.2 parent	0.1^a^added 0.04^a^agreement	2.6 added 0.04 agreement	
		8–15 years					0.03^a^ added 0a agreement		
		16–19 years					0.4^a^ added 0.1^a^ agreement		
Kashani et al. 1987 N = 150	Missouri 1986 DSM-III	DICA Lifetime 14–16 years Parent & adolescent	0.7	0.7	N/R	N/R	0.7 0^b^	0.7 2%^b^ (all cyclothymia)	
Lewinsohn et al. 1995 N = 1,709	Oregon 1988 DSM-III-R/DSM-IV	K-SADS Lifetime 14–18 years Adolescent	6.7^c^	0.1	0.8	6.7	0.1	1.0	
Costello et al. 1996 N = 1,015	Nth Carolina 1994 DSM-III-R	CAPA 3 mth 9–13 years Parent & adolescent	0.1 added	0	0.1	N/R	0	0.1 added	
Andrade et al. 2006 N = 619	Hawaii 1994 DSM-III-R	DISC Lifetime 13–21 years Adolescent	1.5	N/R	1.5 “Mania-hypomania”	N/R	Part of 1.4	1.4 “Mania-hypomania”	
Gould et al. 1998 N = 1,285	USA 1996 DSM-III-R	DISC 6 mth 9–17 years Parent & child/adolescent	1.3	1.2	N/R	N/R	Possibly less than 1.2 (“mania” may include hypomania)	1.2	
Kessler et al. 2009 N = 347	USA 2003 DSM-IV	K-SADS, CIDI Lifetime 13–17 years Adolescent	6.3 K-SADS	0.5	2.3	6.6	0.5 K-SADS 1.0 CIDI	6.2 K-SADS 6.6 CIDI	

^a^Includes BD-I plus BD-II

^b^ Carlson & Kashani (1988) reviewed data concluded 3 adolescents had cyclothymia

^c^ BD-NOS cases of 5.7% failed to continue as bipolar cases in young adult follow-up (Lewinshohn et al. 2000)

### Reanalysis of the eight new studies used by Van Meter et al. (2019a) meta-analysis  sults

The 19 surveys were largely unsuitable for meta-analysis because the methodologies varied in instrumentation, ages of subjects, concordance between informants, prevalence period, and diagnostic criteria. There was wide variation in the reported prevalence rates. BD prevalence rates were zero or close to zero in surveys of prepubertal children.

The eight new surveys covered by Van Meter et al. (2019a) are illustrated in Table 2 and narrative comments follow.

**Table 2:** The eight extra epidemiological studies reported on by Van Meter et al. (2019a)

Source Subjects	Location Year completed Criteria	Instrument Prevalence period Age	Informant Critique	Van Meter et al. (2019a)			Parry et al. (2020) – this paper		
				BD-I %	Undifferentiated BD-I %/BD-II %	Total Bipolar Spectrum %	BD-I %	Total Bipolar Spectrum %	
Päären et al. 2014 N = 2,300	Sweden 1991-1993 DSM-III-R	BDI-C CES-DC Attempted Suicide DICA-R-A Lifetime 16–17 years	Youth report only. Methodology does not allow for accurate community prevalence. Two stage screening with depression questionnaires followed by a diagnostic interview for hypomania.	0.04	-	4.0	1/2,300 = 0.04% fulfilled criteria for a manic episode.	Hypomania spectrum: 90/2,300 = 3.9%; (full 1.7%, brief 0.8%, sub-syndromal 1.4%). Total bipolar spectrum = 3.94%	
							Adult follow-up 15 years later: Sole adolescent BD-I case not reported. 2/64 (3%) adolescent hypomania spectrum developed mania/adult BD-I. 3/130(2%) adolescent MDD developed mania/adult BD-I.	Adult follow-up 15 years later: 4/64 (6%) adolescent hypomania spectrum had hypomania/BD-II in adulthood. Total bipolar spectrum = 9% of this cohort. 13/130 (10%) adolescent MDD had hypomania/BD-II in adulthood. Total bipolar spectrum = 12% of this cohort.	
Tijssen et al. 2010 N = 1,395 or 705	Germany 1994 Follow-up 1996, 2002 DSM-IV	DIA-X/M-CIDI Lifetime 14–17 years	Youth report only. 37 cases in 1,395 identified as at least 4 days hypomanic/manic lifetime symptoms, but these excluded from follow-up cohort of 705, as study focused on development of new symptoms.	-	2.7	14.3	Not defined	37/1,395 = 2.65%	
								8-year follow-up of cohort of 705 adolescents excluding 37 original total bipolar spectrum subjects: No further episodes hypomania/mania emerged, many manic and depressive symptoms, authors don't define cases.	
Roberts et al. 2007 N = 4,175	Texas, USA 2000 DSM-IV	DISC-IV 12 mth 11–17 years	Youth report only for diagnosis. Divided results according to whether impairment criteria of DISC-IV or CGAS were applied or not.	0.4	1.2	-	0.39 (with/out impairment) 0.31 (DISC impairment) 0.22 (CGAS impairment)	1.2 (with/out impairment) 0.31 (DISC impairment) 0.31 (CGAS impairment)	
Kozloff et al. 2010 N = 5,673	Canada 2002 DSM-IV	CIDI Lifetime 15–24 years	Youth report only. Diagnoses on DSM-IV criteria but more liberal duration criteria of “several days or longer”.	2.0	-	-	Not defined	All ages 15–24 years 15–18 years 19–24 years	3.0
									2.1
									3.8
Anselmi et al. 2009 N = 4,452	Brazil 2005-2006 DSM-IV/ ICD-10	DAWBA [Estimated prevalence reads as cases in diagnostic phase. Formula in stats section] 11–12 years	Child plus mother informants combined with psychiatrist adjudication where discrepant.	0	-	-	0	0	
Pan et al. 2014 N = original sample = 9,937; final sample = 1554 high-risk + 958 random-selection = 2512	Brazil 2009 screening 2010-2011 DSM-IV	DAWBA Lifetime 6–12 years Parents of 9937 6–12-year-old children interviewed Family History Survey. A sample of 2512 random + high-risk children selected for parent interview DAWBA.	Parent/caregiver only informant. ‘Exuberant’ hypomanic symptoms not associated with impairment or psychopathology. ‘Under-control’ hypomanic symptoms overlap with ADHD and ODD/ CD.	-	0.2	1.8	0.2 (BD-I/ BD-II) 0.2^a^	1.8 1.4^a^	
Vizard et al. 2018 N = 9,117	England 2017 ICD-10	DAWBA Lifetime 2–19 years	2–4-year-old (parent) 5–10-year-old (parent + teacher) 11–16-year-old (parent + youth + teacher) 17–19-year-old (parent + youth)	0.1	-	-	Not defined^b^	0.0 (5–16-year-olds) 0.1 (17–19-year-olds) NB by age/gender the two groups with cases were 11–16-year-old boys (0.1%)^c^ and 17–19-year-old girls (0.3%)	
Karacetin et al. 2018 N = 5,842	Turkey	K-SADS-PL Lifetime 2014–2015 Turkish school year 7–10 years	Parent only	0	0	0	0	0	

^a^“Weighted prevalence” – personal communication Pan-Parry (2019)

^b^ICD-10 F-30/F-31 codes include “mania/hypomania/BD-I/BD-II/BD-undifferentiated”

^c^Personal communication Ford-Parry (2019) indicated “youngest case bipolar spectrum disorder was 16-years-old”

#### Päären et al. (2014) Conducted 1991–1993, follow-up 2006–2008, Sweden

This Swedish study screened 2,300 16–17-year-olds for lifetime depression. Three hundred and fourteen who screened positive then participated in the Diagnostic Interview for Children and Adolescents (revised for DSM-III-R, adolescent version, DICA-R-A) for hypomanic symptoms (“hypomania spectrum”). Additionally, 317 controls who had not screened positive for depression were also assessed with the DICA-R-A. The combined and enriched (not strictly community) cohort of 631 adolescents’ findings were: “90 participants with hypomania spectrum (40 full-syndromal, 18 with brief episode, and 32 subsyndromal), 197 participants with major depressive disorder (MDD) and 229 controls” (p. 4).

Fifteen years later, at ages 31–33 years-old, the cohort was diagnostically reviewed with the Mini International Neuropsychiatric Interview Plus (MINI Plus) as well as screening for personality disorders. The authors found: “continued mood disorder in adulthood (MDD, bipolar disorder, or dysthymia) was reported by 60.9% of the hypomania spectrum group and 70.0% of the MDD group” (p. 9). Although many had been prescribed antidepressants, just 7% of hypomania spectrum (n = 4) and 2% of MDD and no controls had been prescribed antipsychotics. No hypomania spectrum and just 2% of MDD had been prescribed anticonvulsants and nobody prescribed lithium.

In a separate article Päären et al. (2013) reported that of the 64/90 adolescents at follow-up who had reported lifetime hypomanic syndromes at baseline, 38 adults (59%) reported an adult MDD, but only four (6%) reported recurrence of hypomania and only two (3%) reported a manic episode. The authors stated it was unlikely that hypomania spectrum adolescents lost to follow-up had converted to BD as the register data showed “only a few received inpatient or outpatient care for mental disorders” (p. 196) and concluded: “The results indicate that only a small proportion of adolescents with hypomania spectrum episodes continue to have (hypo)mania in adulthood” (p. 190). In comparison, for the 130/197 adolescents with MDD, three (2%) reported manic episodes and thirteen (10%) reported hypomanic episodes at follow-up at ages 31–33 years-old (p. 194). Both groups had similar rates of adult MDD (56% and 55% respectively) and of no adult mood disorder (33% for both). Thus, the rates of conversion to BD-I or BD-II disorder in adulthood from adolescent hypomania spectrum compared with MDD episodes was similar.

#### Tijssen et al. (2010) Conducted 1994–2002 (follow up over 8 years) Germany

This German study interviewed 1,395 14–17-year-old adolescents on four occasions over eight years with the World Health Organisation’s German version Composite International Diagnostic Interview (DIA-X/M-CIDI) to derive lifetime DSM-IV diagnoses and subsyndromal mental symptoms. Minimum duration criteria was 2 weeks for depressive symptoms and 4 days for hypomanic/manic symptoms. At first follow-up, a parent was interviewed for family psychiatric history and whether ADHD diagnosed, otherwise interviews were with the adolescents.

The study focussed on risk factors (presence of family psychiatric history, negative life events, substance use, ADHD diagnosis and personality factors) related to the new appearance of “subclinical expression of bipolar psychopathology” (p. 255). Therefore, all 37 (2.65%) of cases with hypomanic/manic episodes were excluded after the baseline interview. Additionally, any participants (n = 653) with less than full data were excluded.

Of the remaining 705 participants, 162 (23.0%) had a lifetime history of subsyndromal hypomanic/manic symptoms at baseline and 125 (17.7%) subsyndromal depressive symptoms. The three follow-up interviews examined for onset of new hypomanic/manic symptoms or depressive symptoms and then persistence of such symptoms and relationship to the risk factors. It appears from Tijssen et al.’s article that although many of the 705 subjects had manic symptoms, Tijssen et al. do not report if any developed a full hypomanic/manic episode over the 8-year follow-up.

As there was no follow-up data on the 37 (2.65%) of the original 1,395 at baseline who recorded a lifetime hypomanic/manic episode of > 4 days duration, it is unclear how many of this group might have had recurrent episodes in the 8-year follow-up period, which could have been a means of validating true BD cases.

The authors postulated that the transitory nature of subsyndromal hypomanic/manic symptoms and a link with novelty seeking temperament in adolescence that faded by young adulthood represented physiological dopaminergic mechanisms of adolescent development. Tijssen et al. (2010) concluded: “This hypothesis would fit well with the observed high prevalence of manic symptoms reported in adolescents that are transitory for the great majority of individuals” (p. 263).

#### Roberts et al. (2007) Conducted 2000, USA

This study utilised data from a representative sample of 4,175 youths in Texas aged 11–17-years-old and one of their caregivers. The DISC-IV was used to examine for 12-month prevalence of DSM-IV diagnoses. Impairment was measured using the DISC-IV impairment scale and the Child Global Assessment Scale (CGAS).

The authors only used youth reports for making DSM-IV diagnoses, adding caregiver reports to ascertain level of impairment. They state: “we did not interview parents about the DSM-IV disorders assessed by youth interview”, noting that “many studies have demonstrated considerable discordance in parent–child reports of psychopathology” and “there is little consensus on how parent and youth reports should be combined in epidemiologic studies” (p. 7).

The findings were divided according to DISC-IV without impairment, with DISC-IV impairment criteria, or with CGAS impairment criteria. They found a 12-month prevalence of all DSM-IV disorders of 17.1% (no impairment), 11.1% (DISC-IV impairment) and 5.3% (CGAS impairment). The respective findings for mania were 0.39% (95% C.I. 0.18–0.61), 0.31% (0.12–0.51), 0.22% (0.05–0.39) and for hypomania were 0.81% (0.50–1.12), 0%, 0.09% (0–0.20). The youngest (11–12-year-olds) had roughly half the odds ratios of having a mood disorder than both the 13–15-year-old and 16–17-year-old cohorts, but hypomania/mania were not distinguished from MDD/Dysthymia.

#### Kozloff et al. (2010) Conducted 2002, Canada

This study extracted data from the representative Canadian Community Health Survey: Mental Health and Well-being (CCHS 1.2) survey of 36,984 people aged 15-years and older. The CCHS 1.2 used the World Mental Health-Composite International Diagnostic Interview (WMH-CIDI) based on DSM-IV criteria. Caregivers were not interviewed. Kozloff et al. looked at data from the 5,673 participants aged 15–18-years-old and 19–24-years-old.

The CCHS 1.2 used DSM-IV criteria for diagnosis of a lifetime manic episode but rather than full 7-day duration criteria, defined a manic episode of lasting “several days or longer” (p. 351). Kozloff et al. presumed that:

[T]he sample likely included subjects with [BD-I] as well as BD-II and ‘not otherwise specified’ but the CCHS 1.2 interview did not include criteria required to accurately differentiate these subgroups of BD (p. 351).

Kozloff et al. found the “overall weighted lifetime prevalence rate of BD was: 2.1% (1.4–2.7) among adolescents 15–18-years and 3.8% (3.0–4.6) among young adults aged 19–24” (p. 352). There were high lifetime comorbid anxiety disorders (46.6%) and 12-month problematic substance use prevalence (50.4%).

In their discussion the authors noted these rates of BD meant that “BD in youth is a fairly common, highly comorbid disorder” (p. 353) and higher than in older adults. They postulated that attrition from suicide and misadventure may reduce rates of BD in older adults, but also listed study limitations that may have over-estimated the BD rate in this youth and young adult sample: 1) the CCHS 1.2 survey used a more liberal duration criteria than strict DSM-IV; 2) the WMH-CIDI was not calibrated for adolescents and “may not accurately distinguish BD from other disorders [such as] ADHD and conduct disorder [which are] more common in adolescents”; 3) episodes may be difficult to distinguish from “extreme and frequent mood swings” that “adolescents tend to experience more than adults”; 4) “the CCHS 1.2 survey relied on self-report without the support of collateral information” (p. 353).

#### Anselmi et al. (2009) Conducted 2005–2006, Brazil

This study involved children from a Brazilian 1993 Birth Cohort Study. Anselmi et al. screened 4,452 preadolescents (mean age 11.3-years-old) and their mothers with the Strengths and Difficulties Questionnaire (SDQ) in 2004/2005 and later conducted a diagnostic phase of interviews with the children (mean age 12.4-years-old) and mothers using the Development and Well-Being Assessment of Children and Adolescents (DAWBA) in 2005/2006. Prevalence period was not stated but based on citation (Goodman et al. 2000) was 1 month for ‘emotional disorders’.

All who screened positive above an a-priori severity of SDQ symptoms (n = 122), and a random selection of those below the cut-off (n = 158) for a total of 280 children and their mothers, were interviewed using the DAWBA. Diagnoses were based on both parent and child report with adjudication by a child psychiatrist author where parent interviews were discordant.

Extrapolating, Anselmi et al. (2009) calculated: “479 preadolescents out of the 4,448 participants of the 1993 birth cohort would present at least one psychiatric disorder according to either the ICD-10 or DSM-IV” (p. 138). The main findings were: any DSM-IV/ICD-10 diagnosis 10.8% (95% C.I. 7.1–14.5); any anxiety disorder 6.0% DSM-IV/6.2% ICD-10; any DSM-IV/ICD-10 depressive disorder 1.6% (0.4–3.6); ADHD/hyperkinetic disorder 4.1% (1.6–6.4) DSM-IV/2.7% (0.9–5.0) ICD-10; DSM-IV/ICD-10 oppositional-conduct disorder 4.4% (1.6–6.4); eating disorders 0.1% (0.3–0.5); and tic disorders 1.3% (0.2–2.2).

There were no cases of hypomania/mania reported.

#### Pan et al. (2014) Conducted 2009–2011, Brazil

This Brazilian study screened 9,937 parents of 6–12-year-old children using the Family History Survey (FHS) and then interviewed 2,512 subjects comprised of “a high-risk subgroup (n = 1,554) and a random-selection group (n = 958)” (p. 626) with the parent-version DAWBA. Impairment was measured using the SDQ impact score.

Pan et al. found 479 (19.1%) of the 2,503 subjects screened positive for lifetime manic symptoms and five children (0.2%), mean age 9.4 ± 1.34 years, met criteria for lifetime BD-I/BD-II, while 41 subjects (1.6%) met criteria for BD-NOS.

Other psychiatric disorders lifetime prevalence rates for the whole sample of 2,503 were: any disorder 25.7%; any anxiety disorder 9.9%; any depressive disorder 2.9% ; ADHD 10.9%; any CD/ODD 6.8%. For the 479 screened positive for lifetime manic symptoms the comorbidity was higher: any disorder 43.6%; any anxiety disorder 18.4%; any depressive disorder 6.3%; ADHD 19.8%; any CD/ODD 11.9%.

Pan et al. also examined two dimensions of manic symptomatology: 1) an ‘under-control’ subscale including irritable, distractible, risk taking, less self-control, poor concentration, invades other people’s personal space, bossy, less concerned about getting into trouble, overly sexed, constant changes of plans and activities, flight of ideas, talking to strangers, overconfident, and restless; 2) an ‘exuberant’ subscale including cheerful, joking and laughing more than usual, outgoing, active, fast talk, noisier, gets more done, full of energy, excitable, and restless. Only the ‘under-control’ subscale was associated with psychiatric morbidity and psychosocial impairment. The authors commented that the ‘exuberant’ episodic manic symptoms being mild and not associated with impairment may need to be severe and frequent to be of significance. They noted that “several under-control symptoms of mania are also symptoms of ADHD and ODD/CD. Therefore, we may have ascertained symptoms of externalizing disorders rather than manic symptoms” (p. 631).

They also commented on the similar methodology and findings to Stringaris et al. (2010) (one of the original 12 epidemiological studies), who found very low rates of BD-I/BD-II and a larger group of BD-NOS children/youth who may or may not progress to BD proper. In their contribution to the *CAMH* debate on PBD, Pan et al. (2019) state:

BD-NOS prevalence was 1.6% in the Brazilian sample, compared to 1.1% by parent report and 1.5% by youth report in the British B-CAMHS (Pan et al. 2014; Stringaris et al. 2010). We have also found that overall BD prevalence was 1.8% in the [Brazilian] study, exactly the same prevalence rate reported in Van Meter et al. (2011) metanalysis. This result adds to the pool of non-US studies in which youth BD could be identified using both narrow (0.2%) and broad (1.6%) criteria. However, this finding does not necessarily mean that all these subjects are ‘true’ bipolar cases. (p. 104).

In the same article they add:

[U]ntil we do fully understand the pathophysiology of BD, impairment may help guide our judgement when we face the hard task of distinguishing manic symptoms from normative (and perhaps developmentally essential) exuberant and under-controlled behaviour in youth. (p. 104)

#### Vizard et al. (2018) conducted 2017, England

This UK study surveyed 9,117 2–19-year-old children and youth using the DAWBA. Informants (Vizard et al. 2018, p. 6) were parent for 2–10-year-olds; parent, teacher and child/youth for 11–16-year-olds; and parent and youth for 17–19-year-olds. Trained lay interviewers conducted the interviews and clinician raters made the ICD-10 diagnoses.

The study did not distinguish between types of BD due to the small numbers and reported zero cases (to first decimal place) amongst all 5–17-year-olds. However, by gender and age group the prevalence rate was 0.1% amongst 11–16-year-old boys (no girls) and 0.3% amongst 17–19-year-old girls (no boys) (NHS Digital 2018).

Prevalence rates for 7,654 children and youth in the 5–19-year-old range then were (with 95% CI): All: rate of 0.0% (0.0 – 0.1); boys: 0.0% (0.0 – 0.1); girls: 0.1% (0.0 – 0.2). In comparison the rates for any anxiety disorder was: All: 7.2% (6.6 – 7.9); boys: 5.4% (4.7 – 6.2); girls: 9.1% (8.1 – 10.1). For any depressive disorder: All: 2.1% (1.7 – 2.5); boys: 1.4% (1.0 – 1.8); girls: 2.8% (2.2 – 3.5). However, disorders increased with age, for instance 20.3% and 6.5% of older adolescent girls were recorded as having an anxiety or depressive disorder respectively. These rates could be considered low in comparison to some other nations. The authors note a limitation was the response rate of only 52% to the stratified random selection across England.

#### Karacetin et al. (2018) conducted 2014–2015, Turkey

This study, representing 44 Turkish child and adolescent psychiatry departments, drew upon The Epidemiology of Childhood Psychopathology in Turkey (EPICPAT-T) Study to focus on the prevalence of affective disorder in preadolescent children. The subjects were 5,842 primary school students (mean age 8.7 ± 1.2-years). Parents were interviewed with the Kiddie Schedule for Affective Disorders and Schizophrenia for School Age Children-Present and Lifetime Version (K-SADS-PL). The authors noted that the K-SADS-PL covered the full gamut of bipolar spectrum disorders. They did not discover a single case.

They attributed the lack of bipolar spectrum disorders to the younger age of the cohort, comparing it to the absence of mania in the Great Smoky Mountains Study in the US (Costello et al. 1996) and stated: “Consistent with these findings, there were no cases of BP disorder in our study sample, in which school-age children with a mean age of 8.7 ± 1.2 years were included” (p.519).

Karacetin et al. noted that “irritability can be a part of many psychiatric disorders” and that “the prevalence of ADHD in the EPICPAT-T Study was found to be 12.4% … some of the cases with BP might have been misdiagnosed as ADHD” (p. 519). The prevalence of depressive disorders in this study was (with/without impairment): All depressive disorders 2.5%/1.6%; major depressive disorder 1.06%/1.7%; dysthymia 0.2%/0.2%; adjustment disorder with depressive features 0.4%/0.17%; depressive disorder NOS 0.15%/0.14%.

## Discussion

### Heterogeneity and meta-analyses

Van Meter et al. (2019a) attributed the increase of community prevalence of PBD of 1.8% to BD spectrum disorders in pediatric age range of 3.9% to:

[T]he previous meta-analysis combined rates of bipolar I, bipolar II, and bipolar-NOS to estimate an average prevalence rate, the rate of 3.9% estimates the rate of bipolar spectrum disorders, not a mix of definitions. (p. e3)

However, some of the large newer studies reported zero or near zero rates of BD and this was reflected in the updated meta-analysis finding “the pooled rate of bipolar I was 0.6% (95% CI, 0.3%—1.2%)” (p. e1), just half the 1.2% rate for BD-I reported in the 2011 meta-analysis.

They also reported the epidemiological studies’ findings were statistically heterogenous for both bipolar spectrum disorders (Q = 759.82, df = 32, P < 0.0005) and for BD-I (Q = 154.27, df = 13, P < 0.0001). Despite this marked heterogeneity, the first two of three headlined ‘clinical points’ (Van Meter et al. 2019a: p. e2) spoke of constancy and uniformity in PBD prevalence rates across time and geography:

- The prevalence of pediatric bipolar disorder in the community has been relatively constant over time.
- The prevalence of pediatric bipolar disorder is not higher in the United States than it is in other countries.

Their third ‘clinical point’ acknowledged the heterogeneity:

- Knowledge about the community prevalence of pediatric bipolar disorder is limited by the lack of studies from non-Western countries, the inconsistency in measurement across studies, and the small number of studies that include prepubescent youth.

We concur with Van Meter and colleagues on this third comment. There is a need for consistency in measurement and there is insufficient data on prepubescent youth, although three of the newer eight studies (Pan et al. 2014; Vizard et al. 2018; Karacetin et al. 2018) add important data on preadolescent children.

### Discrepant informant data

In their discussion, Van Meter et al. (2019a) address the discrepant informant reports. This was highlighted in our 2018 reanalysis. A prime example was the Dutch study (Verhulst et al. 1997) where there was zero concordance as to the presence of BD-I/BD-II between youth (1.8%) and parent (1.2%) report. In their original meta-analysis, Van Meter et al. (2011) generally took the higher prevalence rates from the original surveys. For example, they added the Dutch youth and parent reports to derive a rate of 2.8%. However, if prevalence was based on parent and youth concordance, then the rate was 0%. In their updated meta-analysis Van Meter et al. (2019a) chose to utilise the parent report (1.2%) rate only.

They chose the parent report only also for the British survey (Stringaris et al. 2010) and have updated the data in their meta-analysis to note the parent report for BD-I/BD-II is just 0.1%, while 1.2% for bipolar spectrum disorder, which combines the 0.1% with the 1.1% prevalence for BD-NOS on parent report. Van Meter et al. (2019a) cite their group’s own research on caregiver, youth and teacher rating scales (Youngstrom et al. 2015) that parent report is more reliable than youth report. Nonetheless, they acknowledge that “best practice guidelines suggest integrating information from multiple informants” (p. e8).

We believe it is important for readers to be aware that there was zero agreement between youth and parent in the Dutch survey. There was similar very low concordance in nearly all the epidemiological surveys with more than one informant. Based on lack of age differential, lack of depressive symptoms and concordance between youth and parent report “no better than chance”, Stringaris et al. (2010) doubted the phenomenology of BD-NOS in their British study was part of a true bipolar spectrum:

[O]ur findings call into question BP-NOS in youth really is a variant of DSM-IV BP; superficially similar symptoms may not necessarily imply deeper similarities in aetiology or treatment response. (p. 36)

In total, eight epidemiological studies had both parent and youth informants. In addition to the Dutch and British studies, four of these studies’ methodologies gave low prevalence rates. The Irish study (Lynch et al. 2006) reported zero cases of bipolar spectrum disorders. The more recent Brazilian study (Anselmi et al. 2010) reported zero cases of BD-I. The US Great Smoky Mountains study (Costello et al. 1996) zero cases of BD-I and a 0.1% 3-month prevalence of BD-II. There was just a single adolescent case of mania for a BD-I rate of 0.7% in the US Missouri study (Kashani et al. 1987) when impairment criteria were accounted for (Carlson and Kashani 1988). In contrast, the remaining two US studies gave high rates by adding parent and child report rates: Gould et al. (1998) found a BD-I rate of 1.2% and Kessler et al. (2009) a BD-I rate of 0.5% (based on K-SADS) or 1.0% (based on CIDI), and total bipolar spectrum rate of 6.3% (K-SADS)/6.6% (CIDI). The level of concordance or kappa value are not revealed, so the validity of these high rates is open to speculation.

### Problems with epidemiological survey diagnostic specificity

By their nature, large epidemiological studies rely on lay interviewers and the level of insight of informants such as parents and youth to gather the raw data that, depending on methodology may or may not be reviewed by a clinician researcher. The interviews usually occur once and are based on verbal report in the absence of mental state examination. This contrasts with diagnoses formed in clinical settings. We agree with Van Meter et al. (2019a) when they state:

[T]he reliance on fully structured diagnostic interviews, common in epidemiologic studies, is likely to result in many people being misdiagnosed; bipolar disorder is challenging to diagnose and clinical judgement is often necessary to establish episodicity and impairment, both of which are important to accurate diagnosis. (p. e8)

Clinical diagnoses, particularly for cases severe enough to warrant inpatient treatment, allow time to assess a patient’s mental state in context and clarify collateral histories from multiple informants. Such history taking can deduce the impact of developmental trauma, child maltreatment, attachment insecurity, family dynamic, school bullying and other stressors on the externalising and internalising symptoms that may at a superficial level sound like BD on a single structured interview focused on phenomenology.

Nevertheless, the discrepant clinical diagnostic rates of BD of several-100-fold for children and younger adolescents between the US and other countries suggests the persistence of what Carlson and Klein (2014) described as the divergent ‘liberal’ and ‘conservative’ perspective on the boundaries of BD in children and youth. These divergent liberal (pro-PBD hypothesis) and conservative (classical view of early-onset BD) perspectives continue in the academic literature as we recently reported (Parry et al. 2019b).

### Bipolar disorder very rare in pre-adolescent children

Van Meter et al. (2019a) note that “older minimum age was associated with higher rates (*P* < 0.0001)” (p. e6). This observation was more robust in the updated meta-analysis as whereas only three of the original 11 epidemiological studies had children under age 12, five of the newer eight studies did.

In two of the original studies (Costello et al. 1996; Gould et al. 1998) age of those who met bipolar spectrum criteria were not given. Neither was age given of the single case of BD-I/BD-II amongst 3,618 8–15-year-olds (0.028%) in the British Stringaris et al. (2010) study, and the authors did not consider this a “definite” (p. 33) case as it lacked parent and youth concordance.

The five newer studies provide more data on the under-12 age group, but still find few cases. The two Brazilian studies (Anselmi et al. 2009; Pan et al. 2014) and the English study (Vizard et al. 2018) all use the DAWBA instrument and thus comparable methodology to Stringaris et al. (2010). Anselmi et al. (2009) found no cases of bipolar spectrum disorder amongst 4,448 11–12-year-old children screened with the SDQ of whom 280 children’s parents were questioned with the DAWBA. Pan et al. (2014) found five cases of BD-I/BD-II among 2,512 6–12-year-olds for a rate of 0.2%, this held true for four cases out of 2,108 for the weighted prevalence. Pan and colleagues concluded it was doubtful as to whether the 42 BD-NOS cases for a 1.7% BD-NOS prevalence were truly part of a bipolar disorder spectrum. The large English study (Vizard et al. 2018) found no cases of bipolar spectrum disorder (ICD-10 F-30/F-31 codes) under age 16. It is also more probable that most of the 10 to 13 cases of (DISC-IV) mania/hypomania among the 4,175 11–17-year-olds in Texas (Roberts et al. 2007) were adolescents. There were zero cases of (K-SADS-PL) bipolar spectrum disorder among the 5,842 Turkish 7–10-year-olds although rates of depressive disorders were low and some false negatives may be obscured by the high ADHD rate as the authors suggest (Karacetin et al. 2018).

It should be noted that the DAWBA, used by the two British and two Brazilian studies, has just a single screening question about “going abnormally high” and that past research has found that strict screening questions in epidemiological studies can underestimate rates of subthreshold and emerging BD (Merikangas et al. 2007). On the other hand the K-SADS-PL used in the large national Turkish study failed to find any bipolar spectrum disorder cases in their pre-adolescent cohort.

### Adolescent hypomania often not resulting in adult bipolar disorder

The majority of bipolar spectrum cases across the 19 studies were of adolescents and by self-report. The validity of these diagnoses is thrown into some question by two of the newer studies (Päären et al. 2013, 2014; Tijssen et al. 2010) that incorporated follow-up interviews some years later. Although the German study of Tijssen et al. excluded baseline lifetime cases of BD, it did not find any new cases of BD over the ensuing 8 years, despite the presence of hypomanic symptoms in some subjects and the study focusing on emerging affective symptoms. The 15-year follow up in the Swedish study (Päären et al. 2013, 2014) found only a small percentage of adolescents with “hypomania spectrum disorder” developed BD-I (3%) or BD-II (6%) by their early 30s. However, these are just two studies and whether they reveal an overdiagnosis of hypomania in adolescence, prolonged remissions or time-limited forms of bipolar spectrum disorder is unclear but is an area warranting further research.

The nature of bipolar spectrum diagnoses in younger Americans was also brought into question by an article titled: “Are there developmentally limited forms of bipolar disorder?” (Cicero et al. 2009). Data from the U.S. National Epidemiological Survey on Alcohol and Related Conditions (NESARC) study showed that as people grew older, decreasingly fewer individuals reported either a 12-month or lifetime prevalence of bipolar spectrum disorders. The decline was steady with advancing age, with 4% of 18–20-year-olds meeting NESARC criteria for a bipolar spectrum diagnosis but only 0.5% of those aged 60 and older.

### Limitations of epidemiological surveys

A limitation of epidemiological surveys is that questions are asked of lay public often by trained lay interviewers about mental symptoms usually without context. The public may understand such questions in diverse ways, affirmative answers might not equate with true ‘pathology’. The problem of making diagnoses based on decontextualized information in such surveys is greater than in clinical settings. This problem limits any narrative analysis of such surveys, but we would argue that it limits statistical meta-analysis of epidemiological surveys even more, as the nuances that can be described by narrative analyses are lost in statistical lumping of diverse data.

### Belief versus disbelief in the PBD hypothesis

Beyond just diverse methodologies and data are the accepted beliefs of researchers and public, particularly with a controversial diagnosis, across different times and places. Van Meter et al. (2019a) stated: “diagnostic criteria are not uniformly applied. Asking the same questions will not lead to the same diagnoses if participants’ responses are interpreted through different filters” (p. e7). We agree. Whether it is wide variation in prevalence rates from epidemiological studies, the massive difference in clinical diagnosis rates or geographic variation in academic research (Parry et al. 2019b), BD in childhood and adolescence remains a controversial area. For a quarter of a century the field has been divided between those adhering to the ‘liberal’ PBD hypothesis of common pre-pubertal onset of atypical mania versus those adhering to the ‘conservative’ classical view that mania/hypomania generally does not present until mid-adolescence or later (Carlson and Klein, 2014).

Our reading of the epidemiological surveys that cover the presence or absence of BD in children and youth is that classical criteria rather than altered criteria for BD in children and adolescents should be applied. Adult BD criteria as per DSM-5 or ICD-11 should be used across the lifespan, as recommended by the British NICE guidelines ((NICE) 2014) and the ISBD PBD Task Force report (Goldstein et al. 2017). This would limit over-diagnosis and iatrogenic adverse effects, which in addition to metabolic and cardiotoxic effects of antipsychotic drugs, include risk of cerebral atrophy (Bastiampillai et al. 2019). In this context Malhi et al. (2020) argued that: “until we know more about bipolar disorder in childhood and have a robust means of diagnosis, use of the term PBD should be abandoned” (p. 549).

### Answers in longitudinal high-risk offspring studies?

Longitudinal studies of high-risk offspring reiterate this conservative versus liberal perspective divide: five studies support the conservative classical view and find BD is preceded in childhood by increased non-specific symptoms of anxiety and sleep disorders, with depressive episodes in early adolescence; while one study supports the PBD hypothesis of pre-pubertal onset of very brief manic episodes (Duffy et al. 2017).

The field might be at an impasse. However, response to lithium perhaps gives close to a bio-marker to resolve the debate: A systematic literature review of lithium trials in children and young adolescents diagnosed with PBD indicated inferior response compared to lithium for classical mania in older adolescents and adults. In contrast superior response from risperidone may reflect non-specific sedating properties in ADHD and other disruptive disorder driven symptoms (Duffy et al. 2018).

Most recently, Duffy and colleagues have argued that the debate has been resolved (Duffy et al. 2020). In a comprehensive narrative review they highlight that the PBD hypothesis which encompassed “chronic irritability and explosive temper in pre-pubertal children with pre-existing ADHD and/or other learning and developmental disorders” has not been supported by “prospective studies of children at high familial risk” of BD. They further conclude that “epidemiological studies of population and hospital discharge data provided evidence that the pre-pubertal bipolar phenotype was largely a US driven phenomenon” (p. 1).

## Conclusion

This re-analysis found that most epidemiological surveys rarely detect BD before adolescence, which is consistent with clinical diagnosis rates in several nations and the findings of most high-risk offspring studies where BD usually begins after mid-adolescence. If BD is very rare in childhood, there is considerable risk of misdiagnosing BD in children who may receive unnecessary treatment that causes iatrogenic harm.

In the two epidemiological studies where follow-up data was available, many cases of hypomania in adolescence did not progress to adult BD, the significance of this finding warrants further research. While early diagnosis of mania/hypomania is vitally important, overdiagnosis in adolescence or milder remitting prognoses for adolescent hypomania need to be considered.

## Data Availability

Derived from the original published epidemiological studies.
